# Automatic registration of a single SAR image and GIS building footprints in a large-scale urban area

**DOI:** 10.1016/j.isprsjprs.2020.09.016

**Published:** 2020-12

**Authors:** Yao Sun, Sina Montazeri, Yuanyuan Wang, Xiao Xiang Zhu

**Affiliations:** a Remote Sensing Technology Institute, German Aerospace Center (DLR), Münchener Straße 20, 82234 Weßling, Germany; b Signal Processing in Earth Observation, Technical University of Munich, Arcisstraße 21, 80333 Munich, Germany

**Keywords:** GIS building footprints, Large-scale, Registration, SAR image, Urban area

## Abstract

Existing techniques of 3-D reconstruction of buildings from SAR images are mostly based on multibaseline SAR interferometry, such as PSI and SAR tomography (TomoSAR). However, these techniques require tens of images for a reliable reconstruction, which limits the application in various scenarios, such as emergency response. Therefore, alternatives that use a single SAR image and the building footprints from GIS data show their great potential in 3-D reconstruction. The combination of GIS data and SAR images requires a precise registration, which is challenging due to the unknown terrain height, and the difficulty in finding and extracting the correspondence. In this paper, we propose a framework to automatically register GIS building footprints to a SAR image by exploiting the features representing the intersection of ground and visible building facades, specifically the near-range boundaries in the building polygons, and the double bounce lines in the SAR image. Based on those features, the two data sets are registered progressively in multiple resolutions, allowing the algorithm to cope with variations in the local terrain. The proposed framework was tested in Berlin using one TerraSAR-X High Resolution SpotLight image and GIS building footprints of the area. Comparing to the ground truth, the proposed algorithm reduced the average distance error from 5.91 m before the registration to −0.08 m, and the standard deviation from 2.77 m to 1.12 m. Such accuracy, better than half of the typical urban floor height (3 m), is significant for precise building height reconstruction on a large scale. The proposed registration framework has great potential in assisting SAR image interpretation in typical urban areas and building model reconstruction from SAR images.

## 1 Introduction

### 1.1 Motivation

**Fig. 1:**
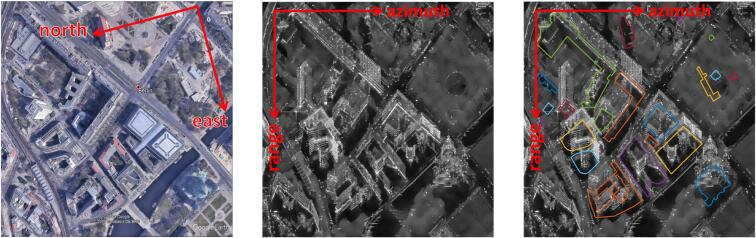
Center Berlin area (the Berliner Dom in the lower-right corner) in a SAR amplitude image with and without GIS building footprints: (left) optical image from Google Earth; (middle) SAR image; (right) SAR image with GIS building footprints superimposed on it.

The increased spatial resolution of modern SAR satellites, such as TerraSAR-X, TanDEM-X, and COSMO-SkyMed, has enabled reconstruction of building models from spaceborne SAR data (Franceschetti et al., 2002, Tupin and Roux, 2003, Guida et al., 2010, Brunner et al., 2010, Sportouche et al., 2011, Liu and Yamazaki, 2013, Zhu and Shahzad, 2014). Such reconstructions are of particular interest to studies concerning areas frequently covered by clouds (Huang et al., 2015) and to applications of disaster response (Brunner et al., 2010, Wang and Jin, 2011), since SAR data are able to provide information regardless of weather and sun illumination conditions. However, object level interpretation from SAR images is still difficult due to the side-looking geometry and complex backscattering mechanism, which introduce phenomena such as layover and shadowing. Furthermore, the increase in spatial resolution complicates the image interpretation, especially in urban areas. For example, Fig. 1 shows central Berlin area in an optical image from Google Earth (left) and in a very high resolution TerraSAR-X image (middle). In the optical image, with the nadir-looking geometry and multi-spectral bands usage, the boundaries of different urban objects are clearly depicted. In the SAR image, with the side-looking geometry and the X-band SAR sensor, the urban structures are clearly visible, but difficult to distinguish from each other clearly.

One branch of SAR image interpretation identifies the link between the SAR image and a corresponding high resolution optical image, such as matching salient features correspondence (Dellinger et al., 2014, Wang et al., 2011, Fan et al., 2012), identifying corresponding patches (Mou et al., 2017), or geometric transformation by 3-D matching (Wang and Zhu, 2019) of optical DEM point clouds and TomoSAR point clouds. However, feature correspondence is difficult to determine in the presence of large geometric differences, while patch-based methods and methods using TomoSAR point clouds usually requires more than 20 SAR images to generate TomoSAR point clouds, and these quantities are often not available. Another emerging method to assist SAR image interpretation is to employ available GIS information, such as building footprint. 2-D GIS data are used by many researchers in urban studies for SAR images interpretation, as they contain direct building shape information. For example, GIS building footprints have been used to identify damages from an earthquake (Dong et al., 2011). Aided by building footprint polygons, it is also possible to estimate building heights from SAR images (Liu and Yamazaki, 2013, Sun et al., 2017) or InSAR data (Thiele et al., 2010). As shown in Fig. 1 (middle) and (right), with the location and shape information of individual buildings from GIS data, the SAR image is more interpretable. The aforementioned studies all require a precise registration of 2-D GIS data and SAR images at a large scale.

### 1.2 Related work and challenges

**Fig. 2:**
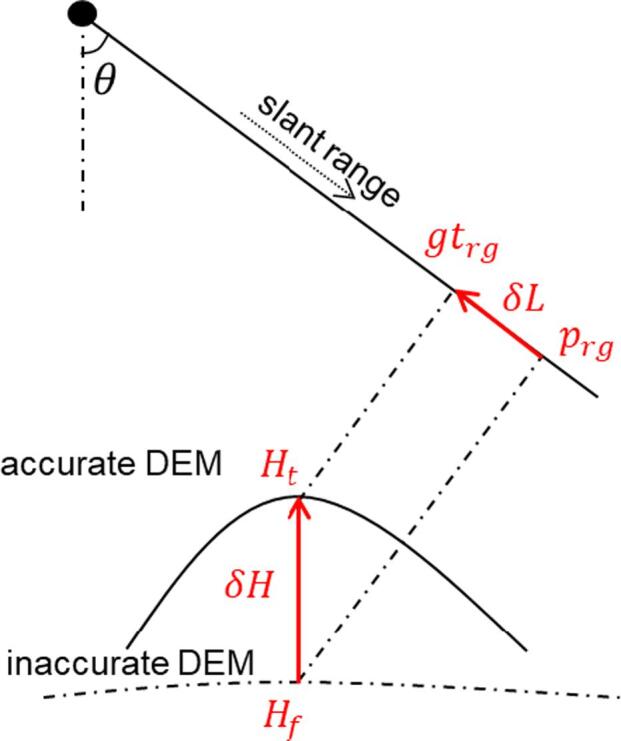
The geocoding error from inaccurate height: $H_{t}$ and $H_{f}$ are the accurate height and inaccurate height of a target point respectively, while ${\mathrm{gt}}_{\mathrm{rg}}$ and $p_{\mathrm{rg}}$ are the corresponding accurate and inaccurate slant range. The height error $\delta H=H_{t}-H_{f}$, and θ is the incidence angle. The resulting error in slant range $\delta L={\mathrm{gt}}_{\mathrm{rg}}-p_{\mathrm{rg}}=\delta \mathrm{Hcos}\theta$.

Precise registration of 2-D GIS data and SAR images at a large scale is often difficult due to lack of the accurate terrain models required in coordinate projection. The projection process of GIS data to the SAR image is referred as radar coding. Fig. 2 illustrates the radar coding error caused by an inaccurate terrain height: $H_{t}$ and $H_{f}$ are the accurate and inaccurate heights of a target point respectively, while ${\mathrm{gt}}_{\mathrm{rg}}$ and $p_{\mathrm{rg}}$ are the corresponding accurate and inaccurate slant range. The height error $\delta H=H_{t}-H_{f}$ causes the range error $\delta L={\mathrm{gt}}_{\mathrm{rg}}-p_{\mathrm{rg}}=\delta \mathrm{Hcos}\theta$, where θ is the incidence angle. For TerraSAR-X, the incidence angle usually ranges from 20° to 55° (Fritz et al., 2008); thus a height error of 10 meters results in a slant range error of 5.73 meters to 9.39 meters. The height error δH is usually inconstant over the observed area by the SAR sensor; hence, so are the range errors.

In addition, the initial alignment after radar coding depends on the geometric accuracy of the two data sets themselves. In general, for object analysis, data with at least meter level geometric accuracy are preferred, since data with low geometric accuracy do not allow detailed analysis, even if they seem to be aligned. The geometric accuracy of SAR images mainly depends on the orbit accuracy and radar timings (Breit et al., 2009), while for GIS data, it mainly depends on the data collecting and processing methods (Zandbergen, 2008). In this work, a TerraSAR-X image with accuracy at centimeters to decimeters level (Breit et al., 2009, Jehle et al., 2008, Balss et al., 2018, Montazeri et al., 2018) and official GIS data with good quality control are used, so that the geometric error of the data is negligible.

Practically, registering the two data sets is challenging. The first challenge is to find the correspondence between them. Due to the geometry difference of the two data sets, objects depicted in one may not be presented in the other. The next challenge is to extract correct features. GIS data consist of buildings boundaries only, however explicit boundary extraction of objects in SAR images is difficult, since the high intensity values are more related to structures and materials than object boundaries. The ambiguity of object boundaries further increases due to the existing speckle noise. In addition, the registration problem is non-rigid, because of the aforementioned inconstant terrain errors. The registration process needs to discover local deformation between the two data sets.

In the research concerned with registering SAR images and GIS data, there are only handful of studies. In Zhu et al. (2015), several building polygons are matched to a SAR image by local adjustment based on the intensity value of the SAR image. However, this only applies for isolated buildings with clear signatures in the SAR images. In Sun et al. (2019), GIS building footprints are registered to a SAR image in a small urban area based on the building correspondence between the two data sets. However, this study does not consider the terrain variation of the urban area, and thus can only be applied in areas with flat terrain.

### 1.3 Contribution

In this work, we propose a framework to automatically register GIS building footprints to a corresponding SAR image in a large-scale urban area.

**Fig. 3:**
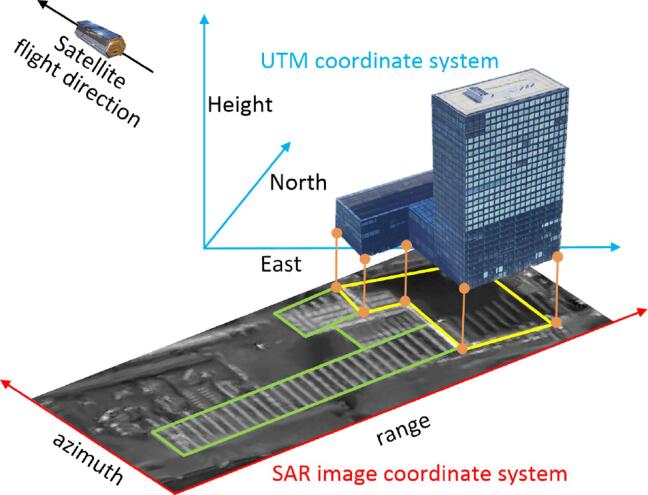
Sketch of the building correspondence between SAR and GIS data: the near-range side of the GIS footprint (yellow polygon) corresponds to the double bounce line in the SAR image, which is approximately the far-range side of the facade signatures (green polygons). (For interpretation of the references to colour in this figure legend, the reader is referred to the web version of this article.)

We exploit building correspondences in the two data sets, as illustrated in Fig. 3. A building in Universal Transverse Mercator (UTM) coordinate system (North-East-Height) and its signature in a SAR image coordinate system (range-azimuth) are plotted: the GIS footprint in the SAR coordinate system is depicted in yellow, while the building signature in the SAR image of the sensor-facing facades are outlined in green. The orange lines connect the near-range side boundary of the GIS polygon and the double bounce lines in the SAR image: they both represent the bottom of the sensor-facing facades and therefore correspond to each other. Based on the correspondence, we extract and register the features from both data sets.

To our knowledge, this is the first time that GIS building polygons and a SAR image have been automatically registered with high accuracy in a large urban area. By exploiting the building correspondence, our method extracts corresponding features. The proposed algorithm registers the two data sets progressively, from the global level to the polygon level. Therefore, it is able to handle the non-rigidity between them.

The registration result has great potential in assisting SAR image interpretation and building model reconstruction from SAR images. Compared to the previous work (Sun et al., 2019), the registration accuracy improves because of two improvements in the workflow: first, the feature extraction in the SAR image is refined by explicitly estimating a bias for each extracted double bounce line; second, the local deformation between the two data sets is considered by progressively registering at three different levels.

In the following, we present the detailed methodology in Section 2 and the experimental results and evaluation in Section 3. Section 4 discusses the results and limitations of the method, as well as some potential applications, which lead to the conclusion in Section 5.

## 2 Methodology

**Fig. 4:**
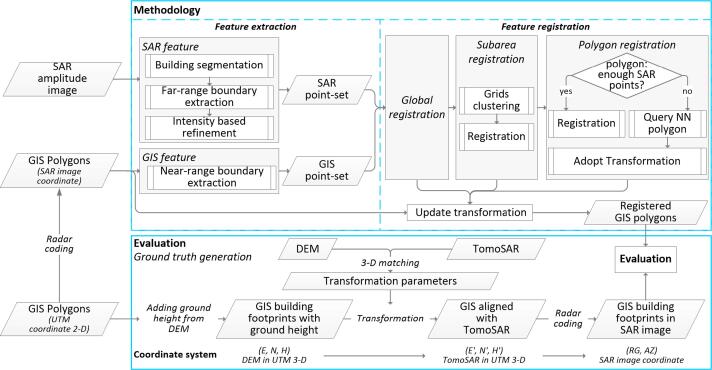
The proposed workflow, containing registration and evaluation. The registration contains two parts: (1) Feature extraction, in which both SAR features and GIS features are extracted and form two feature point-sets; (2) Feature registration, in which the two point-sets are gradually registered, and the transformation parameters are applied to the GIS polygons. The registration results are then evaluated. In the evaluation step, the ground truth is first generated, and the registration result is evaluated by comparing the transformed GIS polygons to their ground truth in the SAR image coordinate system.

Our proposed algorithm consists of two main parts: feature extraction and feature registration, as shown in the methodology block in Fig. 4. In the feature extraction step, the SAR linear features are extracted from the SAR amplitude image and the GIS linear features are extracted from GIS building polygons, then both the linear features are sampled to feature point-sets. In the feature registration step, the two point-sets are registered at the global, subarea, and polygon levels, respectively. After each registration, the transformation parameters are updated and applied to the corresponding GIS polygons.

Since our objective is to register all the GIS polygons to their correct location in the corresponding SAR image, the algorithm is performed in the SAR image coordinate system. Before the main workflow, the GIS data are radar coded to the SAR image coordinate system with heights from a coarse terrain model.

### 2.1 Corresponding feature extraction

#### 2.1.1 Feature extraction from the SAR image: the double bounce lines

In SAR images, the corresponding features are the double bounce lines, which are the bright linear features from signal double bounces at the facade-ground intersections. They cannot be extracted by intensity values only, as other geometries may also appear as bright lines, e.g., regular windows or balconies on building facades, namely, corner lines (Auer and Gernhardt, 2014). However, for one visible facade, its double bounce line is usually located at the far-range side of the parallel corner line group. Therefore, our approach is based on the geometric relationship of the double bounce lines and other corner lines: first, the facade areas are segmented; then the far-range side boundaries of the facade segments are extracted as the preliminary estimates of the double bounce lines; and finally the preliminary double bounce lines are refined by exploiting the intensity of the SAR image. The detailed algorithms are explained below.

**Fig. 5:**
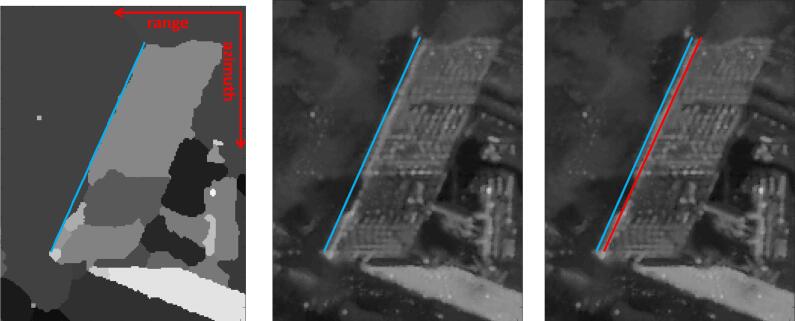
(left) SAR image segmentation result with the extracted far-range boundary of segments (blue) overlaid on it; (middle) SAR amplitude image with the extracted far-range boundary of segments (blue) overlaid on it; (right) SAR amplitude image with extracted far-range boundary of segments (blue) and the double bounce line (red) overlaid on it. Apparently there is a bias between the far-range segment boundary and the double bounce line that needs to be estimated. (For interpretation of the references to colour in this figure legend, the reader is referred to the web version of this article.)
  On SAR images, the corner line repeats itself every one floor of the building. Thus the distance between two corner lines on SAR images is $\mathrm{hcos}\theta /{\mathrm{ps}}_{\mathrm{rg}}$, where *h* is the floor height, θ is the incidence angle, and ${\mathrm{ps}}_{\mathrm{rg}}$ is the pixel spacing of the SAR image in the range direction. For the far-range line $P={⋃}_{i=1}^{n}p_{i},i=1,\ldots ,n$ consists of boundary points $p_{i}$, the bias *s* is estimated:
  (2) $$s^{*}=\arg\;\max_{s}\overset{n}{\underset{i=1}{\sum }}I(r_{i},c_{i}-s),0<s<\mathrm{hcos}\theta /{\mathrm{ps}}_{\mathrm{rg}},s\in N,$$
  where for $p_{i}(r_{i},c_{i})$ at the $r_{i}$-th row and $c_{i}$-th column of the SAR image, $s^{*}$ is the range bias to be estimated, and $I(r_{i},c_{i})$ is the intensity value at $p_{i}(r_{i},c_{i})$. The refined double bounce lines are obtained by adding each bias to the corresponding far-range boundary lines.

A. *Facade segmentation*
  The SAR image is first segmented using the Potts model. The Potts model (Potts, 1952, Geman and Geman, 1984, Mumford and Shah, 1989) formulates segmentation as an optimization problem:
  (1) $$u^{*}=\arg\;\min_{u}\gamma \|\nabla u{\|}_{0}+\|u-F{\|}_{2}^{2},$$
  where *F* is the measured image and the data fidelity is measured by the $L^{2}$ norm. The empirical model parameter γ>0 controls the balance between data fidelity and the regularizing term. The term *u* is a piecewise constant function, whose discontinuity set encodes the boundaries of the corresponding segmentation. The term $\|\nabla u{\|}_{0}$ denotes the total length of the segment boundaries induced by *u*. The Potts problem is NP-hard. In this study, we adopt the minimization strategy of Storath et al., 2014, Storath et al., 2015, where readers can find the details of implementation.
  The Potts model is unsupervised. Hence the facade segments are selected by the following criteria: (1) the area of segments: the largest segments are excluded as background, and very small segments are excluded to reduce outliers in the subsequent registration procedure; (2) the SAR image intensity in the segments area: the average intensity value in the building segments should be greater than the intensity value in ground area, which is approximately the mean intensity of the image; (3) the shape of segments: for building segments, the near-range and far-range sides should be roughly parallel, which is tested using the correlation coefficient.
B. *Far-range boundary extraction*
  After segments selection, the contours of the segments are extracted. To extract the far-range feature lines, a visibility test is performed on the contour of the building segments using the algorithm described in Section 2.1.2 from the far-range side.
C. *Intensity based refinement*
  Due to the smear effect (Auer and Gernhardt, 2014), the double bounce lines do not perfectly overlay the far-range side contours of building segments, which introduces a bias requiring compensation. Fig. 5 (right) illustrates such a bias, where the red line is the desired double bounce line, and the blue line is the estimated one. Such bias is often systematically shifted towards the far-range direction. Since the double bounce line is often the brightest of its neighborhood, we utilize the intensity profile of the SAR image to estimate the bias.

A SAR point-set is sampled from the extracted double bounce lines.

#### 2.1.2 Features extraction from GIS data: the near-range boundary

We found that the corresponding features in the GIS building footprint that are also visible in the SAR image are the “sensor-visible” edges in GIS polygons, which represent the bottom of the illuminated facade in 3-D.

**Fig. 6:**
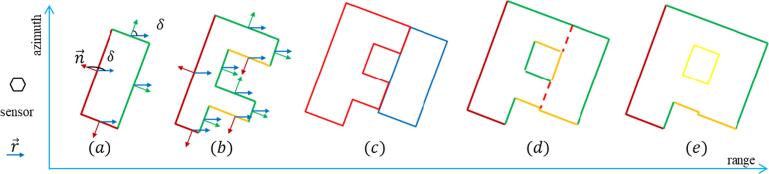
Visibility test: building polygons in a range-azimuth coordinate system (nadir-view). (a) and (b) show two isolated building polygons: $\overset{\to }{n}$ is the outward normal vector of an edge in building polygons, $\overset{\to }{r}$ is the vector in range direction, and δ is the angle between $\overset{\to }{r}$ and $\overset{\to }{n}$. δ∈(90°,270°) holds for the red and orange edges: the red edges are visible, as they are at the near-range side, while the orange edges are partially visible, as they are at the far-range side. The green edges are sensor invisible, as δ∈(90°,270°) does not hold. (c) shows two connected building polygons (red and blue) that should be merged to eliminate the connecting edges (the red dash lines in (d)). (e) shows the merged polygon from (c). The orange edges located at the interior boundary in (d) are partially visible. (For interpretation of the references to colour in this figure legend, the reader is referred to the web version of this article.)

The visibility of polygon edges can be tested via the angle between the sensor line of sight and the edge normal direction. Let $\overset{\to }{n}$ be the outward normal vector of an edge in a GIS polygon, $\overset{\to }{r}$ be the vector in range direction, and δ be the angle between them clockwise from $\vec{r},\;\delta \in [0{}^{\circ},\;360{}^{\circ})$. According to δ and polygon geometry, we have the following three visibility cases, as illustrated in Fig. 6 (a)(b): (1) Visible: δ∈(90°,270°), and the edge locates at the near-range side of the exterior boundary; (2) Invisible: δ∈[0°,90°], or δ∈[270°,360°); (3) Partially visible: δ∈(90°,270°), and the edge locates at the far-range side of the exterior boundary, or at the interior boundary. The partially visible edges are often not visible in the SAR image due to layover and shadow. Therefore, they are not extracted as features. More details are discussed in Section 4.2.

The above visibility test is designed for isolated polygons, e.g., the polygons in Fig. 6(a) and (b). Sometimes, two polygons are connected, e.g., in Fig. 6(c). In such cases, the connected polygons are merged into a single polygon before the visibility test is performed, as shown in Fig. 6(c) and (e).

Finally, the GIS point-set is sampled from the extracted GIS features, which are to be used for registration.

### 2.2 Feature registration

As introduced in Section 1, height error δH causes a range error of δL=δHcosθ. Due to terrain variation, as well as different accuracy of each GIS building footprint, the shift is not constant over a whole city. However, we consider the shift to be constant for individual building instances. Consequently, the registration problem is rigid at the polygon level, but non-rigid at the global level.

However, many building polygons cannot be registered at the polygon level, since there many not be sufficient SAR points to perform a registration. To this end, a three-step registration strategy is proposed: first, a global registration is performed to recover a global transformation that brings the two point-sets as close as possible; second, a subarea registration is performed to recover local transformation for polygons in subareas with similar height error; third, individual polygon registration is performed to recover large local transformation that is not estimated previously. The transformation is therefore rigid at each step, but altogether is non-rigid, since they target different subsets of the whole point-sets. Each registration step is explained in the following subsections.

#### 2.2.1 Global registration

To recover the global transformation, the rigid registration is solved with the Iterative Closest Point (ICP) algorithm (Chen and Medioni, 1992, Besl and McKay, 1992). ICP iterates over two steps: (1) find correspondence set K={(p,q)} from target point-set P and source point-set Q transformed with current translation t and rotation R; (2) update translation t and rotation R by minimizing an objective function E(R,t) defined over the correspondence set K. We use the point-to-point ICP (Besl and McKay, 1992), with an objective:

(3) $$E(R,t)=\underset{(p,q)\in K}{\sum }\|p-\mathrm{Rq}-t{\|}^{2}\text{.}$$

The ICP algorithm is performed to register the GIS point-set to the SAR point-set in SAR image coordinate; thus the SAR point-set is fixed. After calculating the transformation, the GIS polygons are updated by applying the transformation to them.

#### 2.2.2 Subarea registration

In subarea registation, a set of grids is evenly distributed over the whole region. The δH in each grid is assumed to be constant. Therefore, the grid size should be large enough to contain sufficient amount of points in each grid for registration, meanwhile as small as possible to promote the constant δH assumption. In practice, we choose the grid size to be larger than the largest polygon (after merging).

Let *dp* be the distance between one GIS point and its closest SAR point. For all the GIS points and the corresponding SAR points in one grid, the distance set is $D={⋃}_{i=1}^{n}{\mathrm{dp}}_{i}$, where *n* is the number of GIS and SAR point-pairs. If the assumption of constant δH holds, the distribution of *D* will be unimodal with one clear peak center at *C*. In one grid, the point-sets are already registered and no further processing is needed, if C=0; while the point-sets need further registration, if C≠0. To avoid discontinuity of the translation parameters between grids, the connected grids with a similar mode of their distribution *D* are clustered into subareas, using DBSCAN (Birant and Kut, 2007), before performing registration using ICP.

In the same way as in global registration, after subarea registration, the transformation is applied to the corresponding GIS polygons. For the GIS polygons that cross two or multiple subareas, several transformations may be suitable. After each transformation, the polygons are calculated, and the one that permits the smallest point-pair distance is chosen.

#### 2.2.3 Polygon registration

When the distribution of *D* does not show a clear center, the constant δH assumption does not hold. In this case, the registration proceeds to the polygon level, i.e., finding a rigid transformation for each polygon.

For each polygon, there can be two possible situations: (a) There are sufficient SAR points to perform further registration. Then the polygon is further registered using ICP. In practice, we employ the following two criteria to check the feasibility of ICP registration: the ratio of the number of near SAR points and the GIS points should be large, in our experiments larger than 0.7; and the corresponding SAR point shape and the GIS point shape should be similar, in practice a correlation coefficient larger than 0.8 as the threshold. (b) There are not enough SAR points around this polygon, then its nearest neighbor (NN) polygon is queried, and the transformation parameters of the NN polygon is adopted for this polygon. If the polygon has more than one NN polygons, the one that permits the smallest point-pair distance is chosen.

## 3 Experiments

### 3.1 Test site and data

**Fig. 7:**
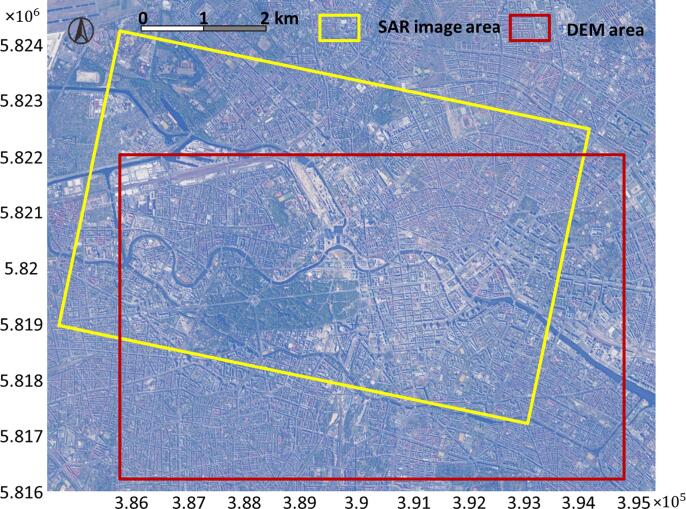
The study area in the UTM coordinate system. The yellow rectangle indicates the area of the SAR image, while the red rectangle is the area of DEM, used as ground truth height. Thus the intersection area of the two rectangles is our test area. (For interpretation of the references to colour in this figure legend, the reader is referred to the web version of this article.)

Berlin is selected as the test site. Frequently monitored using TerraSAR-X data (Zhu and Bamler, 2010, Montazeri et al., 2016, Montazeri et al., 2018, Wang et al., 2017), Berlin is a large-scale urban area containing typical urban forms, such as compact middle-rise area, open high-rise area, open middle-rise area, according to the definition of Local Climate Zones (Stewart and Oke, 2012, Zhu et al., 2019). Our study area is shown in the intersection area of the two rectangles in Fig. 7: the yellow rectangle shows the area of the SAR image, while the red rectangle shows the area of DEM used for ground truth generation. One descending TerraSAR-X image in High Resolution SpotLight mode was used. The incidence angle of the SAR image is 36°, and the heading angle is 194.34°. The pixel spacing of the SAR image in the azimuth direction is 0.871 m, and in the range direction is 0.455 m. The SAR image was filtered using nonlocal InSAR method (Baier et al., 2016). The GIS building footprints data in the study area were obtained from the Berlin 3D-Download Portal (Berlin Partner für Wirtschaft und Technologie GmbH, 2017). After merging, there are 2414 polygons total.

**Fig. 8:**
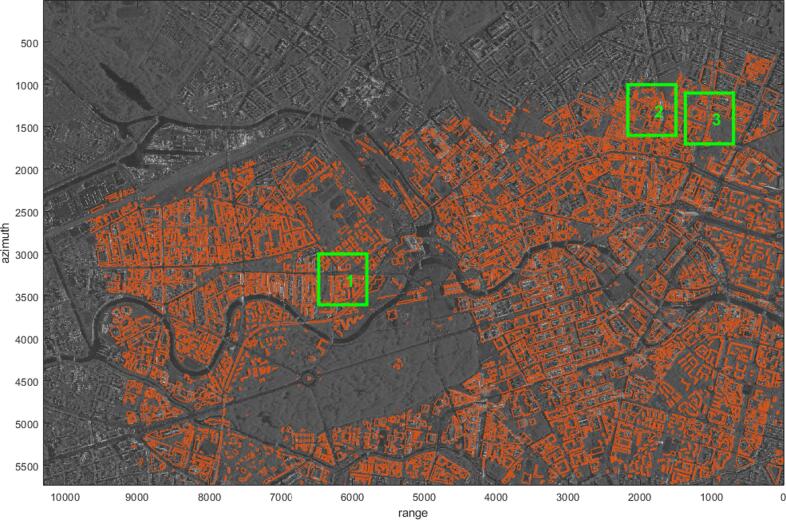
The study area in the SAR image coordinate system. GIS building footprints (red) are radar coded in the SAR image coordinate system with constant height 77.5 m. For detailed inspection of the two point-sets before and after registration, three small areas in the green rectangles are selected: Area 1, 2, and 3 represent areas where registration procedures are needed at global, subarea, and polygon levels, respectively. The point-sets in each area are shown in Fig. 10. (For interpretation of the references to colour in this figure legend, the reader is referred to the web version of this article.)

For radar coding, a constant height value of 77.5 m is added to all the GIS polygons. Fig. 8 shows the radar coded GIS polygons (red) superimposed on the SAR image, and three small areas in the green rectangles are selected to inspect the change of the two point-sets at different steps in the registration procedure (cf. Fig. 10). In the following text, the figures are all shown in the SAR image coordinate system, with the range and azimuth direction the same as in Fig. 8, unless otherwise specified.

### 3.2 Feature extraction

#### 3.2.1 Extracted features from the SAR image

**Fig. 9:**
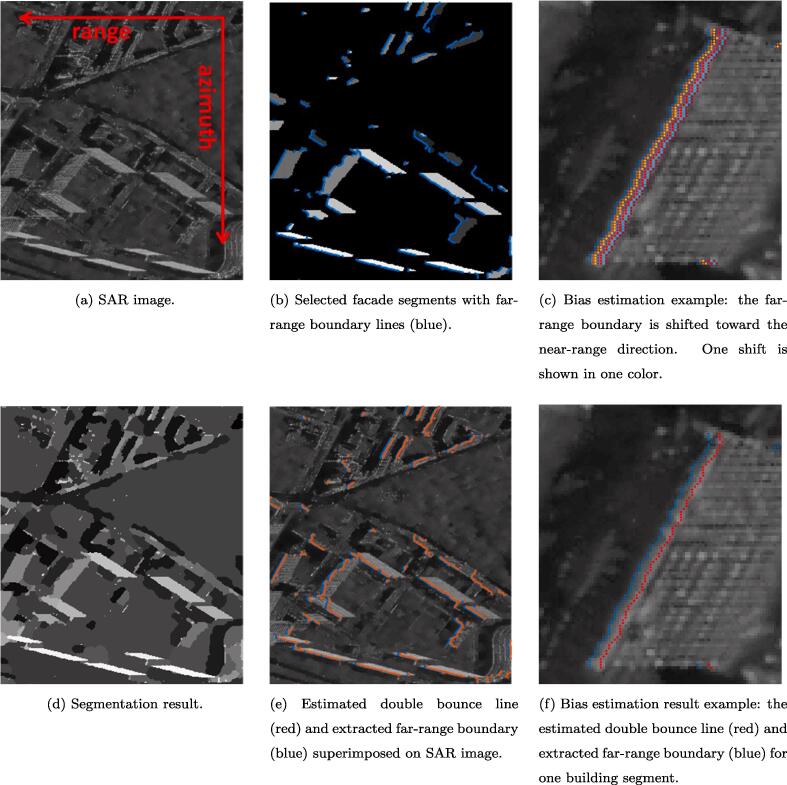
SAR feature line extraction steps. (a) shows an area in a SAR image; (d) shows the Potts segmentation result, and (b) shows the selected facade segments, with the coarse double bounce lines superimposed on the segments; (e) shows the refined double bounce lines in the SAR image; (d) and (f) show examples of the bias estimation process and result.

Fig. 9 shows the steps of feature extraction steps from a SAR amplitude image (Fig. 9(a)). First, the SAR image was segmented using the Potts model: the segmentation results are shown in Fig. 9(d). Second, as shown in Fig. 9(b), the facade segments are selected and the coarse double bounce lines, i.e., the far-range boundaries of the facade segments, are extracted (plotted in blue). Finally, the refined double bounce lines are obtained, and are superimposed on the SAR image in Fig. 9(e), with the coarse double bounce lines plotted in blue for comparison. Figs. 9(c) and (f) show an example of the intensity based refinement of the double bounce line: the coarse double bounce lines in Fig. 9(c) are shifted towards the near-range direction in the given neighborhood; based on the intensity, the red line with estimated bias is chosen, as shown in Fig. 9(f).

**Fig. 10:**
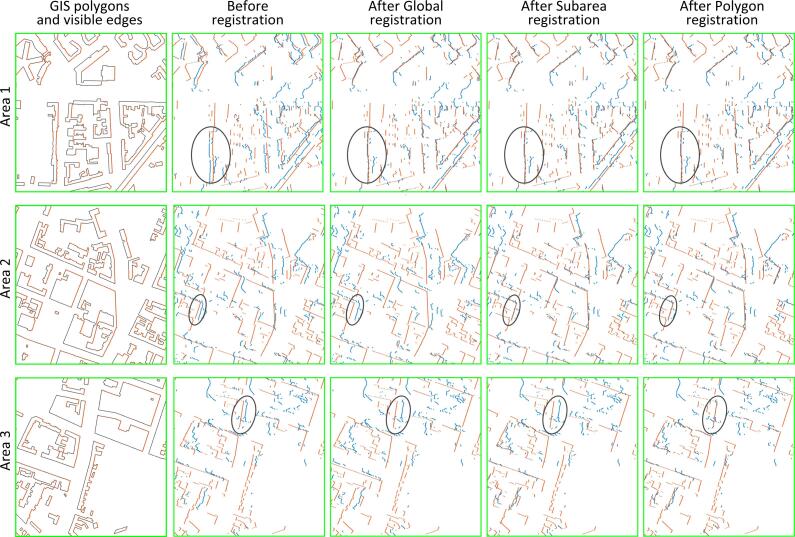
GIS features and registration results of each step in Areas 1–3 of Fig. 8. The first column shows the GIS polygons and the extracted GIS features (red). The second to the last column shows the GIS (red) and SAR (blue) point-sets before registration, after global registration, after subarea registration, and after polygon registration. After global registration, the distance between the two point-sets in Area 1 decreased, but increased in Area 2 and Area 2; after subarea registration, the distance decreased in Area 2; after polygon registration, the distance decr.eased in Area 3. (For interpretation of the references to colour in this figure legend, the reader is referred to the web version of this article.)

In Eq. 2, we assume the height of one floor *h* is around 3 m, hence its neighborhood on the SAR image $hcos\theta /ps_{rg}=3\times cos36{}^{\circ}/0.455=5.33\;(pixels)$. Therefore, we define the bias s∈[1,2,3,4,5,6]. Based on the intensity value, the bias *s* for each far-range line is estimated, and most of them are estimated to be 2 or 3.

#### 3.2.2 GIS features

We extracted the near-range side segments from all the GIS polygons. In Fig. 10, the first column shows that the GIS building polygons from the selected areas are the areas 1–3 marked by the green rectangles in Fig. 8. Their extracted GIS feature lines are plotted in red.

### 3.3 Feature registration

#### 3.3.1 Global registration

ICP was performed on the whole GIS point-set and the whole SAR point-set to determine the global transformation. Since we used a TerraSAR-X image and official GIS building footprints, whose geometric errors are negligible, the registration error mainly comes from the inaccurate height used in the radar coding. Consequently, only errors in the range direction are introduced. Therefore, in our experiments, the rotation matrix R and the translation in the azimuth direction $t_{\mathrm{az}}$ in Eq. (3) were not considered, and the objective was reduced to solve the translation in the range direction $t_{\mathrm{rg}}$.

In Fig. 10, the second column shows the GIS (red) and the SAR (blue) point-sets before global registration, while the third column shows the point-sets after global registration, in the area 1 to 3 in Fig. 8. Details can be seen in areas marked by the black ellipses. As can be seen, after global registration, the distance between the two data sets in Area 1 decreased, while the distance increased in Area 2 and Area 3. This is because the global registration aligns the two data sets to minimize overall distance instead of local distance.

#### 3.3.2 Subarea registration

A set of 16×16 regular grids is defined on the whole region. To contain sufficient points from both the SAR and GIS point-sets, the size of one grid is defined to be larger than the largest GIS polygon (after polygon merging). In each grid area, the distribution of its point-pair distance $D={⋃}_{i=1}^{n}{\mathrm{dp}}_{i}$ is calculated.

**Fig. 11:**
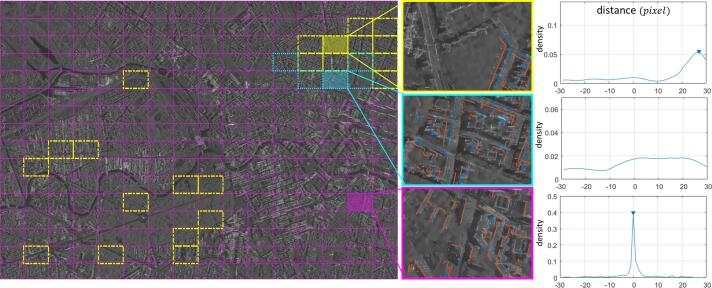
(left) The three types of grids in the study area. Magenta grids: the peak value of the distance distribution is at 0; yellow grids: the peak value of the distance distribution is at *C* (non-zero constant); cyan grids: the distance distribution has no clear peak value. An example of each type is given: (middle) the GIS points (red) and the SAR points (blue) are shown in the grids; (right) the distance distribution between the two point-sets and its peak position are shown in the grids. (For interpretation of the references to colour in this figure legend, the reader is referred to the web version of this article.)

Based on the distribution of *D*, we classify the grid cells into three types, shown in Fig. 11 (left). For magenta grids, the mode of the distribution curve is approximately at 0. Thus no further processing is required. For yellow grids: the mode of the distribution is non-zero. Then adjacent grids with similar distributions are clustered into subareas for subsequent registration. For cyan grids, the distribution has no clear peak. In this case the constant δH assumption does not hold, meaning the polygons need to be examined further.

Fig. 11 provides examples of the three abovementioned types of grids. The distributions of their corresponding point-pair distance are plotted on the right side of Fig. 11: in the magenta grid, *D* has a clear peak at 0; in the yellow grid, *D* has a clear peak at around 27 pixels; however, the cyan grid has no clear peak value.

**Fig. 12:**
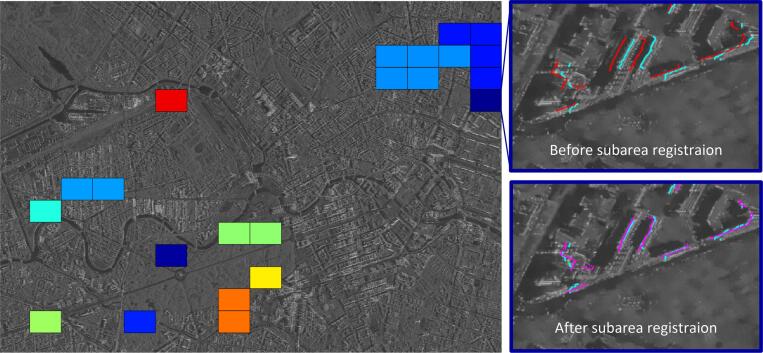
Subareas clustering and registration. The yellow grids in Fig. 11 (left) are clustered into subareas, and each subarea is represented by one color. The GIS and SAR point-sets inside each subarea are then registered. An example before (up) and after (down) subarea registration is shown on the right. The GIS points before (red) are registered to the SAR points (cyan). The transformed GIS points are plotted in magenta. (For interpretation of the references to colour in this figure legend, the reader is referred to the web version of this article.)

Subareas are clustered from the yellow grids, using DBSCAN, as shown in Fig. 12 (left). After subarea clustering, ICP was performed in each subarea. Fig. 12 (right) shows an examples of subarea registration: the GIS and SAR point-sets are well matched after subarea registration.

In Fig. 10, the fourth column shows the GIS (red) and the SAR (blue) point-sets after subarea registration of the three selected areas in Fig. 8. As can be seen, in Area 2, the distance between the two data sets increased after global registration, but decreased after subarea registration, while in Area 3, the distance was still large, so that further registration is needed.

#### 3.3.3 Polygon registration

When the distribution of *D* does not show a clear peak, the constant δH assumption does not hold. The registration proceeds to the polygon level, i.e., we seek to find a rigid transformation for each polygon.

**Fig. 13:**
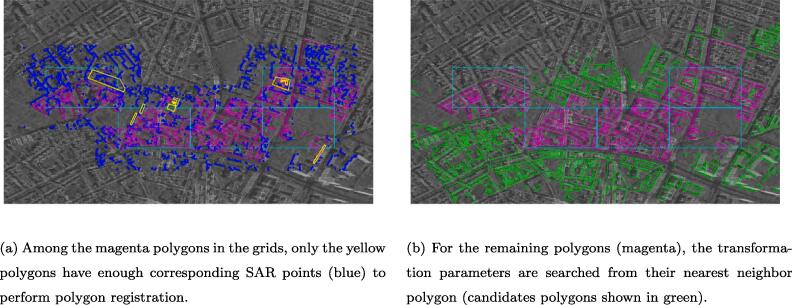
Polygon registration in two cases.

Fig. 13 shows the polygon level registration process in the cyan grids. As shown in Fig. 13 (a), for all the polygons (magenta) in the grids, the ones with enough corresponding SAR points (blue) are further registered, and the polygons after registration are plotted in yellow. In Fig. 13 (b), for the rest of the polygons (magenta), their nearest neighbor polygon is searched from all the candidates polygons (shown in green), and the transformation parameters are adopted.

In Fig. 10, the last column shows the final registration result of the selected areas in Fig. 8. In comparison to the results in previous steps, the distance between the two data sets decreased in Area 3.

### 3.4 Evaluation

#### 3.4.1 Ground truth generation

For evaluation, we generated the ground truth of correctly registered GIS building footprints and the SAR image. Two auxiliary data sets were used: an accurate DEM of the study area and a TomoSAR point cloud produced from the SAR images. The DEM has been created from aerial UltraCam-D^1^ images using the Semi-Global Matching stereo algorithm (Adolf and Hirschmüller, 2011, Hirschmuller, 2008, Hirschmuller and Scharstein, 2009). The ground resolution of the images and the resolution of the DEM are 7 cm/pixel. The TomoSAR point cloud was generated from 109 images using Tomo-GENESIS software developed at DLR (Zhu et al., 2013), and. The DEM provides precise relative terrain heights for radar coding the GIS polygons. The TomoSAR point cloud was used to calibrate the height of the DEM with respect to our InSAR processor, as each point in the TomoSAR point cloud has direct correspondence with the pixels coordinates in the SAR image.

The detailed procedures of ground truth generation can be seen in Fig. 4: (1) we added the height coordinate to the 2-D GIS data footprint using the ground height *H* from the DEM; (2) the 3-D GIS data with coordinates *(East, North, Height)* was registered to the TomoSAR point cloud, using the transformation parameters derived from a 3-D matching of the DEM and the TomoSAR point cloud (Wang et al., 2017); (3) the shifted 3-D GIS data (*East*′*, North*′*, Height*′) were radar coded to the SAR image coordinate system. For the detailed 3-D matching approach of the DEM and the TomoSAR point cloud, readers are referred to Wang et al. (2017).

#### 3.4.2 Evaluation

**Fig. 14:**
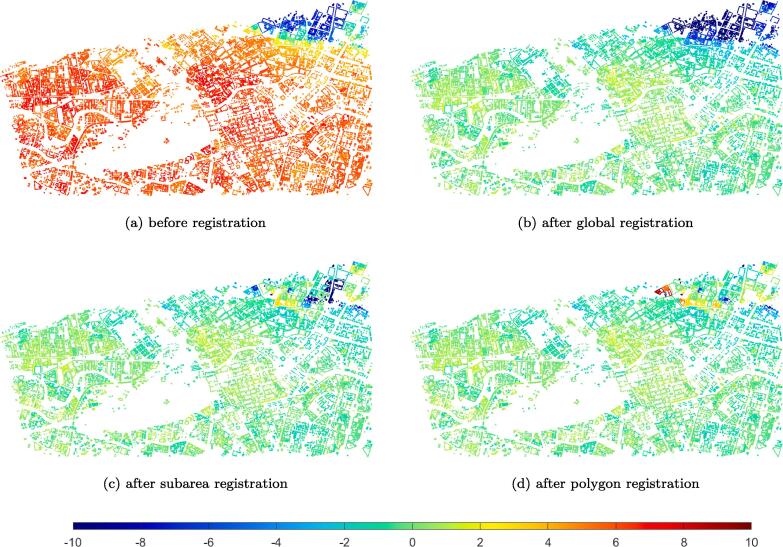
Range error maps of vertices in GIS polygons between registration results and ground truth: (a) before registration, (b) after global registration, (c) after subarea registration, and (d) after polygon registration. Errors are color-coded (meters), in the interval [−10 10]. (For interpretation of the references to colour in this figure legend, the reader is referred to the web version of this article.)

**Fig. 15:**
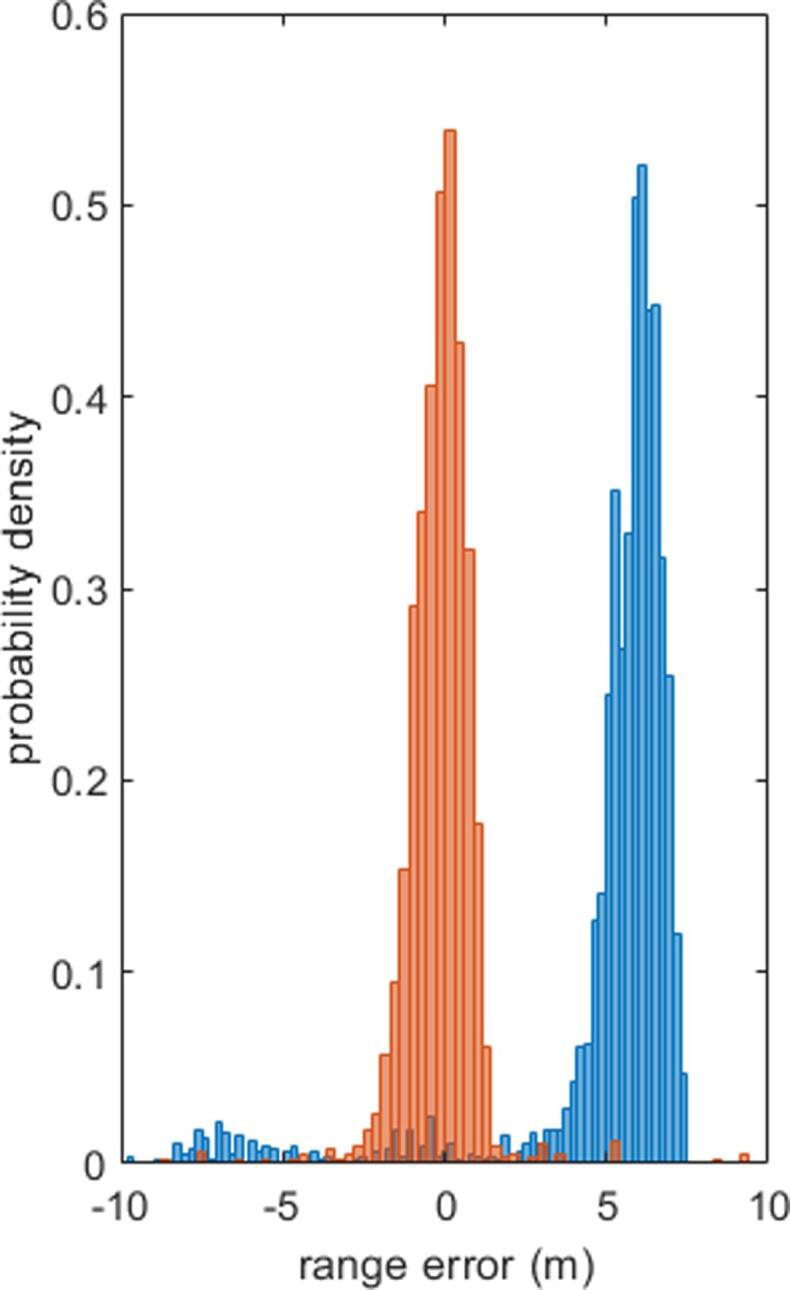
The probability density of vertex distance before (blue) and after registration (orange), compared with ground truth. (For interpretation of the references to colour in this figure legend, the reader is referred to the web version of this article.)

We evaluate the performance of the proposed algorithm using the registration range error δrg. The δrg of each vertex in GIS polygons is shown in Fig. 14, where subfigures (a) through (d) are the error maps before registration, after global registration, after subarea registration, and after polygon registration, respectively. As can be seen, the range difference is not centered at 0 before registration: the majority of δrg is around positive 6 meters, whereas δrg is negative in the upper-right hand corner of the study area. After global registration, this bias is such that the majority of δrg is shifted to 0. Subarea registration and polygon registration further decreases local δrg variations. The final result shows that the average range difference is reduced from 5.91 m to −0.08 m, and the standard deviation of the range difference is reduced from 2.77 m to 1.12 m. The bias and the standard deviation of the errors are listed in Table 1, and histograms of range errors are shown in Fig. 15.

**Table 1:** The bias and standard deviation of the registration errors, comparing the registration results in each step to the ground truth.

	Error (m)	Bias	Standard deviation
	Before registration	5.91	2.77
	Global registration	−0.18	2.77
	Subarea registration	−0.11	1.43
	Polygon registration	−0.08	1.12

## 4 Discussion

In this section, we discuss the potential and the limitations of our algorithm. We also point out some potential applications that may benefit from the proposed GIS and SAR registration framework.

### 4.1 The choice of the terrain model for radar coding

In general, an accurate terrain model for radar coding is preferred, to minimize the range error caused by the height error. However, such terrain models are not available for most areas. For globally available digital elevation models (DEMs), such as the Shuttle Radar Topography Mission (SRTM), the Advanced Spaceborne Thermal Emission and Reflection Radiometer (ASTER) Global Digital Elevation Model (GDEM), and TanDEM-X DEM, the vertical accuracy is often limited (Schumann and Bates, 2018), In addition, the DEMs are surface models instead of terrain models. To acquire terrain heights, a filtering algorithm must be applied. Consequently, the accuracy of the terrain heights is further influenced by the choice of filtering algorithm.

**Fig. 16:**
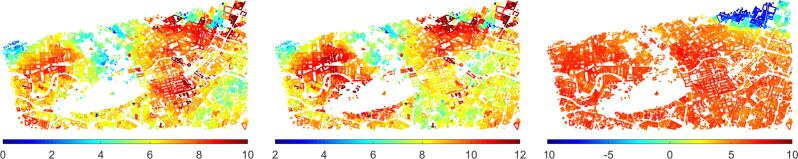
Range error maps comparing to the ground truth, resulting from radar coding using different heights: (left) height from SRTM DEM (median filtered with window size 50 × 50), (middle) height from TanDEM-X DEM (median filtered with window size 50 × 50), and (right) constant height of 77.5 m. Errors are color-coded (meters). Note that the ranges in the three colormaps are different. (For interpretation of the references to colour in this figure legend, the reader is referred to the web version of this article.)

To show the effect of radar coding using different terrain models, we demonstrate in our study area three terrain models for radar coding: a filtered SRTM DEM, a filtered TanDEM-X DEM and a flat terrain model. Fig. 16 (left) shows the range errors over the whole region using filtered SRTM DEM, Fig. 16 (middle) shows the range errors using filtered TanDEM-X DEM, and Fig. 16 (right) shows the range errors using a flat terrain. Obviously, in the case of radar coding using a flat terrain model, the range errors are similar in most areas that can be corrected with global registration. However, in the case of the filtered DEMs, the range error varies more significantly, which increases the difficulty of registration.

Our experience is that a flat terrain model is the first choice for radar coding in urban areas. Since most cities are located in relatively flat areas, the height error is constant for most areas, and the resulting constant range error can be corrected in the global registration step. The residual range errors in non-flat areas are inconstant, and can then be captured and further corrected in the local registration steps. In the presence of a highly varied terrain, a constant height is not enough. A heavily filtered DEM is therefore preferred.

### 4.2 Limitations

#### 4.2.1 Registration errors

Fig. 14(d) shows that after registration, the majority of range differences are around 0. In comparison to Fig. 14(c), in the polygon registration step, the range difference further decreased for most polygons, except for several polygons. The reason is that, these polygons do not have enough nearby SAR points to perform ICP, and thus adopted the transformation parameters of their neighboring polygons. However, these transformation parameters are incorrect if the ground heights of the neighboring polygon differ from the original polygons’ heights. Therefore, these errors are inevitable in such cases.

#### 4.2.2 Applicable scenario, and data selection strategy

**Fig. 17:**
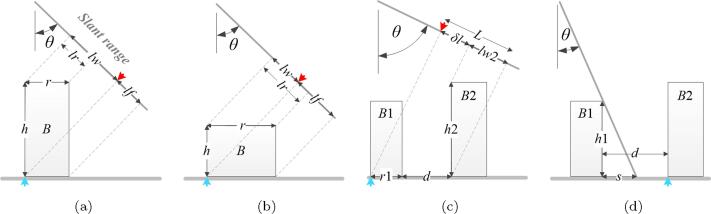
SAR imaging geometry relation of buildings under different settings, where θ is the incidence angle; *h* is the building height, *r* is the building width, *lr*, *lw*, and *lf* are the roof area, the facade area, and the footprint area in the SAR image respectively. The blue arrow marks the bottom of the sensor-facing facade, while the red arrow points at the double bounce line position. (a) and (b) show a single building with different height and width. In (a), the double bounce line is detectable, while it is not in (b). (c) and (d) show two buildings. In (c), the double bounce line of building *B*1 is detectable, because δl>0. In (d), the double bounce line of building *B*2 is detectable, because the distance between the two buildings exceeds the shadow area of building *B*1. (For interpretation of the references to colour in this figure legend, the reader is referred to the web version of this article.)

The proposed method relies on the correspondence between the SAR image and the GIS building footprints. This requires sufficient corresponding features in the two data sets. In SAR images, the double bounce lines are mainly affected by the layover, shadowing areas, which are explained in detail as follows. Fig. 17 illustrates SAR imaging geometries in different situations, where θ is the incidence angle; *h* and *r* are the height and width of building *B*, respectively; *d* is the distance between two buildings; and lr,lw, and *lf* are the layover areas of the roof, facade, and footprint, respectively. The blue arrow marks the bottom of the sensor-facing facade, while the red arrow points to the double bounce line position. We can in general consider the following two cases.

A. Single building: the double bounce line should not be layovered with the building roof.
  Fig. 17(a) and (b) show a building under the same θ, but with different *h* and *r*. Usually the SAR image intensity is low at *lf*, and high at *lr* and *lw*, depending on the number of structures on the roof and the facade. As can be seen, in Fig. 17(a), *lr* lies in *lw*, and so the double bounce line between *lw* and *lf* is detectable. In contrast, in Fig. 17(b), *lw* lies in *lr*, and thus the double bounce line does not show a clear signature when roof area *lr* shows high intensity.
  To ensure that the double bounce line is detectable, *lw* should not be covered by *lr*, i.e., lw⩾lr. Since lw=h·cosθ,lr=r·sinθ, the requirement is therefore: $\frac{h}{r}⩾\tan\theta$.
B. Multiple buildings:
  We simplify this case to two buildings: B1 as the front building and B2 as the rear building, with respect to the sensor. There are two considerations.
  First, the double bounce line of the front building shall not layover with the rear building. As shown in Fig. 17(c), to ensure that the double bounce line of B1 is detectable, it should not be mixed with the layover area of B2, i.e., there must be an area δl>0 with lower intensity between the double bounce line and lw2. Since $\delta l=L-\mathrm{lw}2,\;L=\left(r1+d\right)\cdot \sin\theta ,\;\mathrm{lw}2=h2\cdot \cos\theta$, the requirement is therefore: $\tan\theta >\frac{h2}{r1+d}$ .
  Second, the bottom of the sensor-facing facade of the rear building should not be occluded by the front buildings. As shown in Fig. 17(d), to ensure that the double bounce line of B2 is detectable, B2 should not be occluded by the front building B1, i.e., the shadow *s* of B1 should be no bigger than the distance *d* between B1 and B2. Since s=h1·tanθ, the requirement is therefore: d⩾h1·tanθ.
  Hence, the multiple building case requires: $\frac{h2}{r1+d}<\tan\theta ⩽\frac{d}{h1}$.

The SAR geometries are reflected by the urban morphology of the study area, i.e., the compactness and the building heights, as well as the incidence angle of the SAR image used. Buildings in open areas can be regarded as the single building case, i.e., the constraint is $\frac{h}{r}⩾\tan\theta$. When θ is fixed, study areas with taller buildings are preferred. If most buildings are low-rise or have larger width, SAR images with smaller θ should be chosen. Buildings in a compact area can be regarded as the multiple buildings case, i.e., in addition to $\frac{h}{r}⩾\tan\theta$, the constraints are $\frac{h2}{r1+d}<\tan\theta ⩽\frac{d}{h1}$. When θ is fixed, the average building height in the study area should be small. If most buildings in the study area are high-rise, the SAR image should be chosen so that θ meets the above inequity constraints. However, when $\frac{h2}{r1+d}=\frac{d}{h1}$, there is no solution for θ, meaning that, if the study area is very compact and has many high-rise buildings, the severe occlusion and layover effects prevent the extraction of sufficient double bounce lines. Our method cannot handle this situation.

In practice, 3-D information of study areas is often unknown, so that it is impossible to analyze layover and occlusion for individual buildings. Therefore, pre-knowledge of the urban morphology in the study area is of great benefit, such as a global local climate zone classification map (Zhu et al., 2019).

### 4.3 Further applications

The result of automatic registration of one SAR image and GIS building footprints can be used in data fusion for different applications. We are especially interested in two potential applications.

First, after being registered to a SAR image, the GIS building footprint polygons can be used as iso-height lines in the range direction for object level reconstruction. The iso-height lines can provide shape prior for building height estimation from a SAR image (Liu and Yamazaki, 2013, Sun et al., 2017). The iso-height lines can also be used to group pixels for tomographic inversion using the joint sparsity (Zhu et al., 2015). Second, the registered GIS and SAR data offer the potential of generating large training datasets for building classification. The attributes contained in GIS data can be directly used as ground truth and be learned from SAR image classification. For example, building function can be learned from a large dataset containing SAR images and GIS ground truth labels. Indeed, SAR image classification at the building level is difficult in comparison to using optical images. However, the huge data quantity has potential, especially in areas where cloud coverage limits the use of optical images.

## 5 Conclusion and outlook

In this paper, we proposed a framework for automatic registration of GIS building footprints to a corresponding high resolution SAR image in large urban areas. The facade correspondence between the two data sets is exploited, namely, the near-range side of GIS building polygons, and the double bounce lines in a SAR image originated from the facade-ground intersections. As the transformation is not constant over the whole area due to terrain variation, the registration of the two data sets are performed progressively in three stages: the global level, the subarea level, and the polygon level. Experiments using the TerraSAR-X High Resolution Spotlight image in Berlin confirm that the proposed algorithm effectively reduced the average range distance error from 5.91 m to −0.08 m, and the standard deviation from 2.77 m to 1.12 m.

Despite this promising result, our method also has limitations. Based on the facade correspondence, it requires the double bounce lines of most buildings to be detectable in the SAR image used. To this end, we recommended strategies for handling areas with different urban morphology types. In general, for an open area, areas with taller buildings or SAR images with a smaller incidence angle are preferred; for a compact area, areas with shorter buildings or SAR images with a suitable incidence angle should be chosen. However, in the presence of severe occlusion and layovers between most buildings, our method is not applicable.

In the future, we will apply the proposed method to TerraSAR-X and TanDEM-X stripmap images for level of details (LoD) 1 building model reconstruction.

## Declaration of Competing Interest

The authors declare that they have no known competing financial interests or personal relationships that could have appeared to influence the work reported in this paper.

## Footnotes

1 http://download.microsoft.com/download/2/E/7/2E7FCE24-E085-4A64-B568-25BA956FCB60/UltraCamSpecifications_UCD_UCX_UCXp_UCXpWA_UCL_UCLp.pdf
